# A Quantitative Systematic Review on the Analgesic Efficacy and Adverse Effects of Ketorolac in Third Molar Surgery

**DOI:** 10.3390/clinpract15040081

**Published:** 2025-04-18

**Authors:** Mario Alberto Isiordia-Espinoza, Othoniel Hugo Aragon-Martinez, Nicolás Addiel Serafín-Higuera, Sandra López-Verdín, Eduardo Gómez-Sánchez, Nelly Molina-Frechero, Ronell Bologna-Molina, Juan Manuel Guzmán-Flores, Itzel Joselyn Mora-Falcón

**Affiliations:** 1 División de Ciencias Biomédicas, Centro Universitario de los Altos, Universidad de Guadalajara, Tepatitlán de Morelos 47620, Mexico; jmanuel.guzman@academicos.udg.mx (J.M.G.-F.);; 2 Departamento de Farmacología, Facultad de Medicina, Universidad Autónoma de San Luis Potosí, San Luis Potosí 78290, Mexico; hugo.aragon@uaslp.mx; 3 Facultad de Odontología Mexicali, Universidad Autónoma de Baja California, Mexicali 21040, Mexico; nserafin@uabc.edu.mx; 4 Centro Universitario de Ciencias de la Salud, Universidad de Guadalajara, Guadalajara 44100, Mexicoeduardo.gsanchez@academicos.udg.mx (E.G.-S.); 5 Departamento de Atención a la Salud, Universidad Autónoma Metropolitana-Unidad Xochimilco, Ciudad de México 14387, Mexico; 6 Área de Patología Molecular, Facultad de Odontología, Universidad de la República de Uruguay, Montevideo 11200, Uruguay

**Keywords:** ketorolac, placebo, third molar surgery, postoperative dental pain, meta-analysis

## Abstract

Objectives: This study aimed to determine the number needed to treat (NNT) of ketorolac in comparison to placebo after third molar surgery. Methods: Studies located in PubMed, Scopus, and Web of Science were evaluated with the Cochrane Risk of Bias assessment tool. Data on the onset of analgesia, the number of patients requiring rescue medication, the global or general evaluation of the study medication, and adverse effects were extracted. Data analysis was performed using Review Manager 5.3 software for Windows. Results: The qualitative assessment of the included studies showed that ketorolac was more effective than a placebo and the quantitative evaluation on the onset of analgesia (NNT = 1.6 (95%CIs = 1.4, 1.9), n = 301), the number of patients who took rescue analgesics (NNT = 3.6 (95%CIs = 2.8 to 4.9), n = 563), and the global evaluation of the treatments (NNT = 1.7 (95%CIs = 1.5 to 1.9), n = 475) showed estimates of analgesic efficacy with a statistical difference in favor of ketorolac when compared with a placebo. No statistical difference was observed in adverse effects between ketorolac and placebo (n = 739). Conclusions: There is scientific evidence of moderate quality that allows estimators of the analgesic efficacy of ketorolac to be calculated, which will significantly help the clinician who performs pharmacological treatment after third molar surgery.

## 1. Introduction

In general, the main reasons for performing third molar surgery are dental caries [1], pain and pericoronitis [2], facilitating orthodontic treatment [3], preventing orthodontic failure and/or dental crowding [3], and possible directly associated pathologies, such as dentigerous cysts [4] and ameloblastoma [4,5].

On the other hand, the most common sequelae after third molar surgery in the immediate postoperative period are postsurgical pain, facial swelling, and trismus [6,7,8]. All these inflammatory complications are directly related to age, sex, and surgical difficulty [9] and occur within the first 24 h postoperatively [9,10].

The selection of medications to treat or attempt to prevent complications after any surgical procedure depends largely on the trauma received by the tissues as well as the general health conditions and risks of the patient who will undergo surgery [11,12,13,14,15,16,17]. Proper procedure planning is the key to minimizing these postoperative events [18,19].

Ketorolac, a cyclooxygenase-2 enzyme non-selective non-steroidal anti-inflammatory analgesic (NSAID), is widely used in pre- and post-operative oral surgery [20,21]. Unlike most NSAIDs, ketorolac produces a powerful analgesic effect, while its anti-inflammatory properties are weak [20,22]. Despite the latter, the analgesic potency of this drug seems adequate in most surgical procedures for the extraction of an impacted mandibular third molar [22,23].

Recently, a systematic review and meta-analysis reported the analgesic efficacy of ketorolac compared with other drugs after lower third molar surgery. However, due to the diversity of medications used, it was not possible to calculate specific estimators of analgesic efficacy and safety profile [24]. For this reason, this study was conducted to determine the amount needed to treat and the amount required to harm ketorolac compared to placebo when administered after third molar surgery.

## 2. Material and Methods

### 2.1. Selection Criteria

Inclusion criteria (PICO Strategy) [25]:

Population: Clinical trials comparing ketorolac and placebo after wisdom teeth extraction.

Interventions: Postoperative administration of ketorolac by any route.

Control: Postoperative administration of placebo by any route.

Outcome: The number of patients reporting the onset of analgesia, the number of patients requiring rescue analgesic intake, total number of patients generally rating pharmacological treatments as good, very good, or excellent (global satisfaction evaluation), and adverse effects (nausea, dizziness, vomiting, and headache).

Exclusion criteria:

Trials with more than a 20% loss of follow-up.

### 2.2. Database Search

The PubMed, Scopus, and Web of Science databases were used to locate the title and abstract using the following keywords: “Ketorolac, placebo, and third molar surgery”; “ketorolac, placebo, and third molar removal”; “ketorolac, placebo, and dental extraction”; “ketorolac, placebo, and oral surgery”; and “ketorolac, placebo, and third molar surgical procedure”. Only the “AND” operator was used. In addition, the article type—“Controlled clinical trial” and “Clinical study”— and the language—“English” or “Spanish”—were utilized in the three databases. Studies published until October 2023 were considered in this review. This systematic review was registered by the PROSPERO database (CRD42025649382) of the University of York.

### 2.3. Bias Assessment

The Cochrane Collaboration’s seven-point risk of bias tool was used to assess the risk of bias in each clinical trial. Two independent researchers performed the bias assessment until consensus was reached; the intervention of a third researcher was not required [26,27,28,29].

### 2.4. Data Extraction

The number of patients reporting the onset of analgesia, the number of patients requiring rescue analgesic intake, the total number of patients generally rating pharmacological treatments as good, very good, or excellent (global satisfaction evaluation), and adverse effects—nausea, dizziness, vomiting, and headache—were extracted. Two researchers independently performed information searching, risk of bias assessment, and data extraction. The differences between both evaluators led to the involvement of a third researcher until a consensus on a particular situation was reached [27,28,29].

### 2.5. Statistical Analysis

Data analysis was performed using Review Manager 5.3 software for Windows (University College London, UK). The Mantel–Haenszel test, the Odds Ratio (OR), and the 95% confidence intervals (95%CIs) were used to estimate the effect [26,30]. The determination of heterogeneity was performed using the I^2^ test [31]. The fixed effects model was used when I^2^ was <30, and the random effects model when I^2^ was >30 [31]. Data on rescue analgesia and overall treatment assessment were analyzed overall and by subgroups according to treatment and route of administration. A *p*-value of <0.05 for the overall test and an OR > 1 were considered a statistical difference.

The NNT and 95%CIs were calculated for the onset of analgesia, the number of patients needing rescue analgesics, and the overall evaluation of the treatments using the risk reduction calculator (University of Illinois, Chicago, IL, USA) [32].

## 3. Results

### 3.1. Database Search and Evaluation of Bias

**Figure 1:**
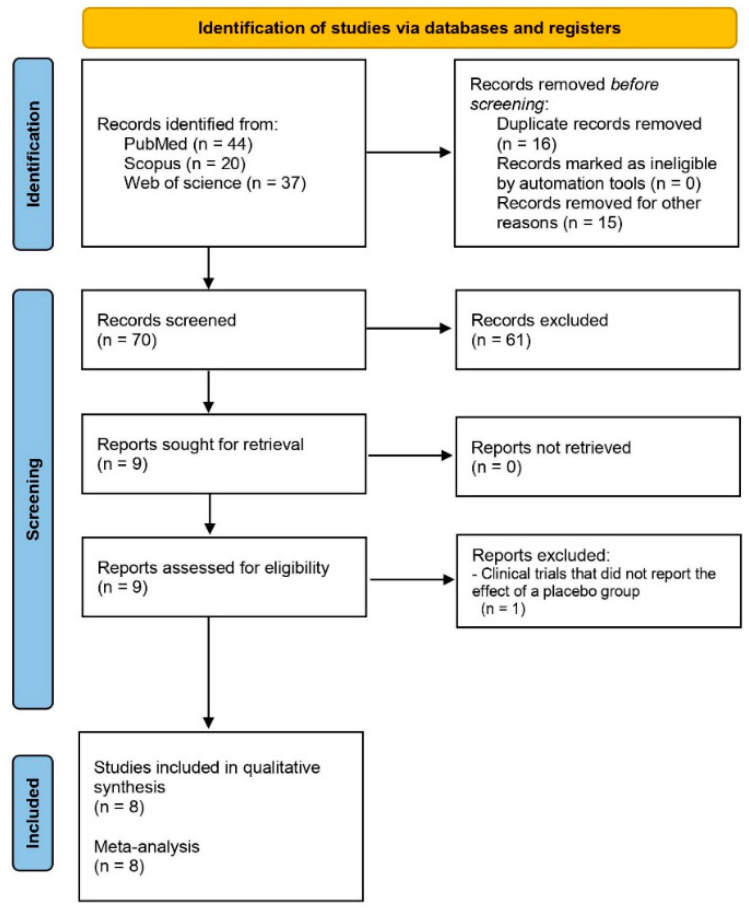
Study flow.

A total of 101 reports were identified in PubMed, Scopus, and Web of Science using the different keywords. After applying the filters mentioned in the search section of the Materials and Methods section, this number was reduced to 70 clinical studies. However, only eight reports were clinical trials comparing ketorolac and placebo after third molar surgery (Figure 1) [33,34,35,36,37,38,39,40].

**Figure 2:**
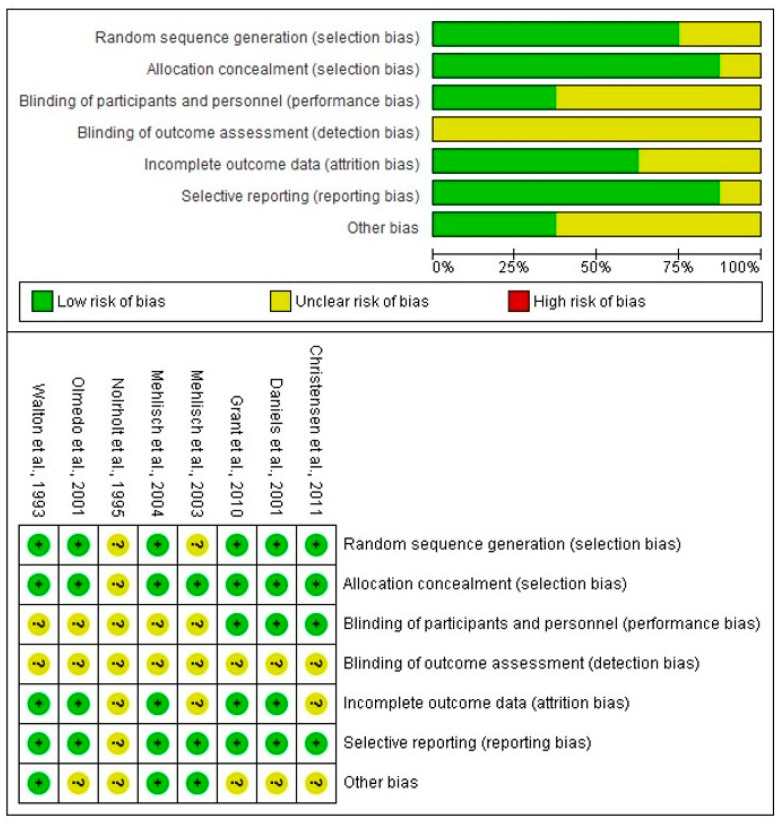
Bias risk assessment [33,34,35,36,37,38,39,40].

The risk of bias assessment showed that the studies evaluated had an unclear risk in all domains. However, it is important to mention that no study complied with point four of the bias assessment tool, which corresponds to blinding of outcome assessment. In all cases, it was classified as unknown (Figure 2) [33,34,35,36,37,38,39,40].

### 3.2. Qualitative Evaluation

All clinical trials had a parallel design [33,34,35,36,37,38,39,40], six studies evaluated postoperative pain after administration of a single dose of the treatments [33,34,35,36,37,38], and two investigations did so through a multiple-dose approach [39,40]. Four clinical trials used ketorolac 30 mg [33,36,37,40], two clinical investigations used ketorolac 10 mg [38,39], and ketorolac 20 mg [39], 31.5 mg [35], and 60 mg was used in only one study [34]. Oral, IM, IV, and nasal administration routes were used [33,34,35,36,37,38,39,40]. Only two studies reported the use of saline as a placebo [34,37], while six clinical trials did not report this information [33,35,36,38,39,40]. The age of the participants ranged between 16 and 65 years of age, with an indication for surgery of at least one lower third molar [33,34,35,36,37,38,39,40]. The most commonly used drug as a rescue analgesic was paracetamol alone [38,39,40] or combined with an opioid [36,37]; one study used ibuprofen [33], and two clinical trials did not report the use of a rescue analgesic [34,35]. Finally, the evaluation period was from 8 h to 9 postoperative days [33,34,35,36,37,38,39,40].

### 3.3. Quantitative Assessment

**Figure 3:**
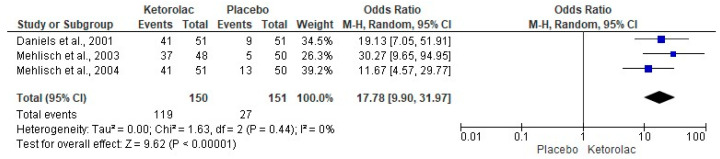
Onset of analgesia [34,36,37].

The onset of analgesia was evaluated with three clinical trials (n = 301) [34,36,37]. The onset of analgesia was reported by a greater number of patients who received ketorolac compared to those patients who were administered a placebo (I^2^ = 0%, OR = 17.78, 95%CIs = 9.9 to 31.97, *p* = 0.00001, Figure 3) [34,36,37]. In addition, the NNT showed that it is necessary to treat 1.6 (1.4 to 1.9) patients with ketorolac to achieve a case of success on the onset of analgesia that would not have occurred with a placebo.

**Figure 4:**
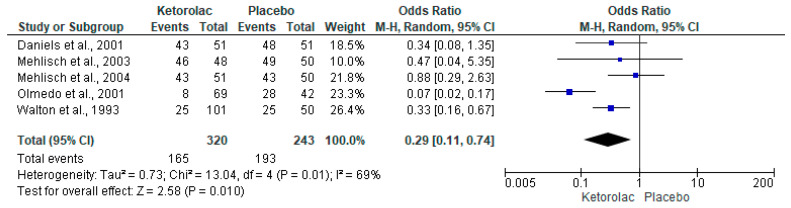
Number of patients who took rescue analgesics [34,36,37,39,40].

**Figure 5:**
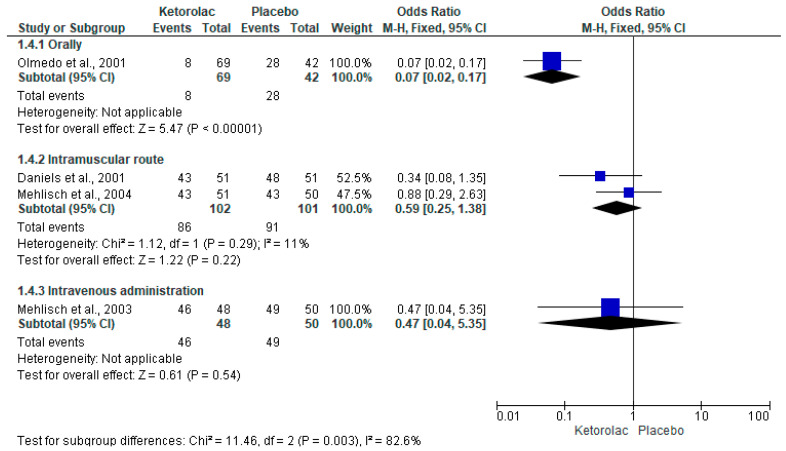
Number of patients who took rescue analgesics according to the administration route [34,36,37,39].

The rescue analgesic intake was assessed using five clinical trials (n = 563) [34,36,37,39,40]. Statistical analysis with pooled data showed that the number of patients who consumed rescue analgesics postoperatively was lower in the ketorolac group when compared to the placebo group (I^2^ = 69%, OR = 0.29, 95%CIs = 0.11 to 0.74, *p* = 0.01, Figure 4) [34,36,37,39,40]. Furthermore, the NNT of the rescue analgesic intake demonstrated that it is necessary to administer ketorolac to 3.6 (95%Cis = 2.8 to 4.9) patients to obtain a successful case in the clinic, which would not have occurred with placebo administration. Pooled analysis by subgroups of administration routes in patients receiving rescue analgesia showed no statistical difference when comparing ketorolac and placebo administered intramuscularly (n = 203, Figure 5) [34,36,37,39].

**Figure 6:**
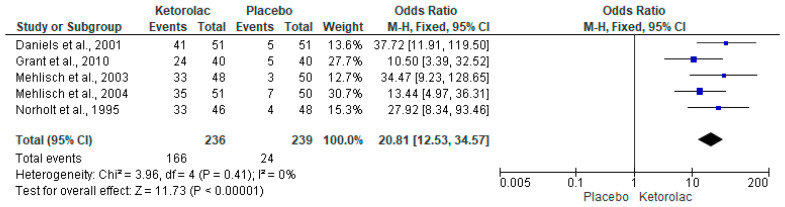
Global evaluation of study medications [34,35,36,37,38].

**Figure 7:**
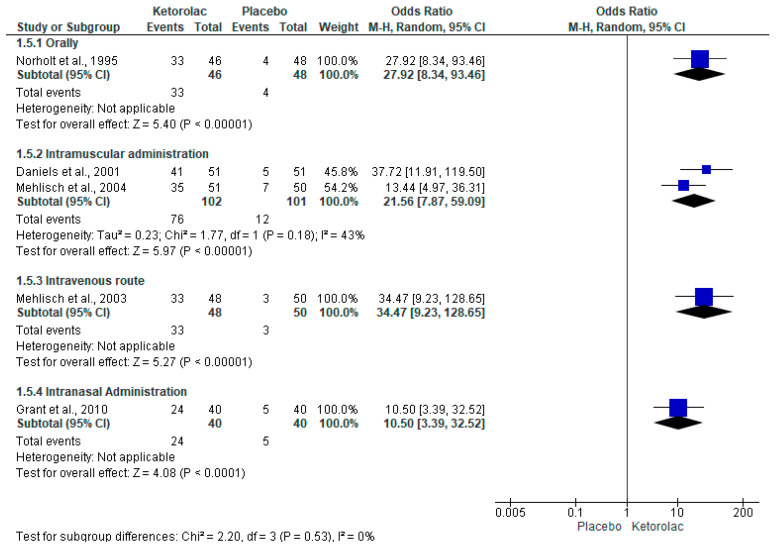
Global evaluation of study medications according to the administration route [34,35,36,37,38].

The global evaluation of the treatments (patient satisfaction with the assigned treatment) was performed with five clinical trials (n = 475) [34,35,36,37,38]. The results of the meta-analysis show that approximately 70% of patients receiving ketorolac reported a “good, very good, or excellent effect” compared to only around 10% of patients receiving a placebo (I^2^ = 0%, OR = 20.81, 95%CIs = 12.53 to 34.57, *p* = 0.00001, Figure 6) [34,35,36,37,38]. Moreover, the NNT indicated that 1.7 (95%CIs = 1.5 to 1.9) patients must be treated with ketorolac for a clinical case to be successful (a patient who reports that the treatment is “good, very good, or excellent”) and that would not have been achieved using a placebo. Subgroup analysis of the overall treatment assessment showed a statistical difference in favor of ketorolac compared with placebo when administered intramuscularly (I^2^ = 43%, OR = 21.53, 95%CIs = 7.87 to 59.09, *p* = 0.00001, Figure 7) [34,35,36,37,38]. In this sense, the NNT showed that it is necessary to treat 1.6 (95%CIs = 1.4 to 1.9) patients for a successful case to occur, which would not have occurred with a placebo.

### 3.4. Adverse Effects

**Figure 8:**
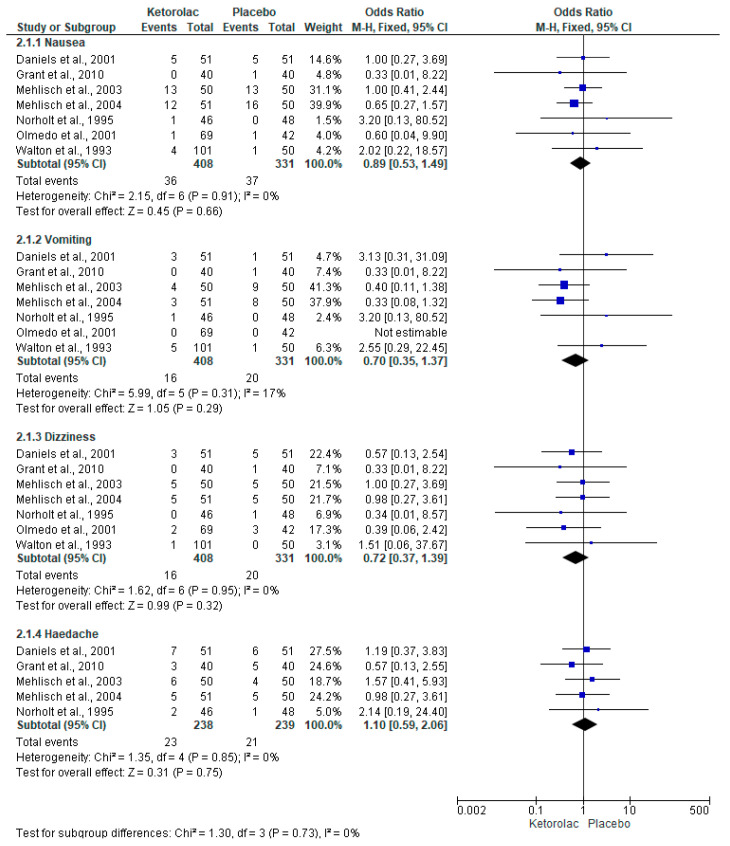
Adverse effects of ketorolac and placebo [34,35,36,37,38,39,40].

The evaluation of adverse effects was carried out with seven clinical trials (n = 739), and the statistical difference was not observed in any of the four adverse effects evaluated (Figure 8) [34,35,36,37,38,39,40].

## 4. Discussion

This quantitative systematic review was conducted based on and in accordance with PRISMA guidelines [41,42]. The most important finding of this systematic review and meta-analysis is the calculation of clinical utility estimators—the NNT and 95%CIs—of the postoperative analgesic efficacy of ketorolac in third molar surgery. These estimators showed a low NNT (close to or less than 3) and within their own 95%CIs, which confirms the statistical difference and provides information that clinicians should consider for the critical and evidence-based use of ketorolac [26]. The NNTs obtained from the assessment of the analgesic efficacy of ketorolac in this study indicate that for the number of patients taking rescue analgesics, 3.6 (95%CIs = 2.8 to 4.9) patients would need to be treated with ketorolac for one patient not to rescue analgesics, which would be considered a clinical success that would not have occurred with placebo. The NNT for the pooled analysis by route of administration of patients who took rescue analgesics was not calculated because no statistical difference was found. The overall assessment of the treatments showed that 1.7 (95%CIs = 1.5 to 1.9) patients would need to be treated with ketorolac for one patient to report the treatment as good, very good, or excellent—a clinical success—which would not have occurred in patients receiving placebo. The NNT from the subgroup analysis of the global assessment of intramuscularly administered treatments showed that 1.6 (95%CIs = 1.4 to 1.9) patients needed to be treated for one patient to rate ketorolac as a good, very good, or excellent treatment, which would not have occurred with placebo. In addition, subgroup analysis showed that oral, intramuscular, intravenous, and intranasal administration of ketorolac produced similar overall assessments and, in all cases, the number of patients who indicated in the overall assessment that the active treatment was “good, very good or excellent” was always higher than that of the placebo group. However, pooled analysis was only possible for the intramuscular route with a limited number of studies; more clinical trials comparing ketorolac and placebo using these same routes of administration are needed to increase the sample size and statistical power.

The present study compared the clinical efficacy of ketorolac with placebo after third molar surgery, allowing for the determination of the analgesic effect of this drug compared to a universal comparator, placebo [43,44]. The results of this study will enable indirect comparisons of the clinical efficacy of ketorolac with the results presented in other publications similar to this one using different drugs. It is important to note that a meta-analytic study was recently published reporting the analgesic efficacy of postoperative ketorolac use compared with other drugs after third molar surgery. However, in that study, it was not possible to calculate the NNT and CIs for ketorolac and the treatments with which this drug was compared [24].

McNicol et al., 2021 performed a systematic review and meta-analysis on the analgesic efficacy and safety of postoperative ketorolac use in different types of surgeries [45]. The authors concluded that ketorolac provided an adequate analgesic effect compared to a placebo. That study calculated the estimators of the clinical utility of ketorolac obtaining NNT and 95%CIs similar to ours. However, it is essential to remember that this study included different types of surgeries [45] and, therefore, does not provide specific information on the analgesic efficacy of ketorolac in lower third molar surgery. Smith et al., 2000 performed a systematic review and meta-analysis on the efficacy of pethidine, morphine, and ketorolac in various types of surgeries. The statistical analysis of that study showed that ketorolac 10 mg had an NNT greater >5 while the analgesic efficacy increased with a higher dose; namely, the NNT of ketorolac 30 mg was 3.4 (2.5 to 49) [46]. However, an upper 95%CI = 49, such as that obtained in the study by Smith et al., 2000 [46], indicates a lack of precision in the estimate, which makes it difficult to extrapolate the results to the population studied [26,30,47,48,49]. In our meta-analysis, the NNT and the 95%CIs of the number of patients who required analgesic rescue and the global evaluation of the treatments were close to or less than 3. In the clinical variables evaluated, it could be assumed that ketorolac is a highly effective drug when used to control postoperative complications in oral surgery [26,50,51].

McNicol et al., 2021 conducted a systematic review and meta-analysis evaluating the adverse effects of the postoperative administration of intravenous ketorolac in different types of surgery. The authors reported that the adverse effects of ketorolac on the nervous and gastrointestinal systems were only slightly superior to those produced by placebo. On the other hand, they highlighted that serious adverse effects with the use of this drug are infrequent and comparable to those observed with the use of a placebo [45]. These authors also performed another systematic review and meta-analysis on the analgesic efficacy of ketorolac in pediatric patients. They reported that adverse effects were overall similar between ketorolac and placebo [52]. In this study, the overall evaluation of the adverse effects of ketorolac and placebo showed no statistical differences. Given this evidence, we can consider that ketorolac is a drug with a low risk of adverse effects. There were also no serious adverse effects reported for ketorolac in any of the clinical trials included in this systematic review.

The most important advantages of this quantitative systematic review were the approach of evaluating the analgesic efficacy of ketorolac and a placebo in a single type of procedure—third molar surgery—with data from randomized clinical trials with unclear risk of bias, a similar methodology among these studies that allowed working with results, in most cases, with low heterogeneity and having performed a robust statistical analysis, with a relatively large sample size, and using powerful and conservative statistical methods. A positive point that we must highlight is the calculation of the NNT and the 95%CIs—which had not been calculated previously—as estimators of analgesic efficacy that have direct implications in the clinical use of ketorolac. Conversely, this study had several significant limitations: the small number of scientific articles (randomized clinical trials) included in this systematic review and meta-analysis, which limited the sample size; all studies included showed an unclear risk of bias; thus, we advise the reader to approach the results of this review with caution. Due to the few clinical trials that met the selection criteria, subgroup analyses based on different doses could not be conducted, and even the subgroup analysis by route of administration included a small number of studies, making pooled analysis impossible in most instances; when it was feasible, it involved a sample size smaller than that used for the overall assessments. Incorporating studies in additional languages and broadening the search to additional databases would have enhanced the number of clinical trials featured in this systematic review, enlarged the sample size, strengthened statistical power, and minimized the risk of bias. Another limitation of our study relates to the number of surgeries performed in each case. Some only performed the extraction of one-third molar, while others reported the extraction of one to four third molars, which influences the intensity of postoperative pain experienced by the patient. Patients who underwent four surgeries will experience greater pain intensity compared to those who underwent only one surgical extraction.

## 5. Conclusions

The results of this systematic review indicate that ketorolac was consistently more effective than placebo in relieving postoperative pain following mandibular third molar surgery. Despite its limitations, this study consolidates the best available evidence in the international literature, and the results are supported by sound methodology and robust statistical analysis, allowing for more reliable conclusions than those obtained in individual studies.
